# Has COVID-19 affected the publication productivity of neurosurgeons in UK and Republic of Ireland? A bibliometric analysis

**DOI:** 10.12688/healthopenres.13445.2

**Published:** 2024-08-12

**Authors:** Hariss G Paremes Sivam, Jigi Moudgil-Joshi, Chandrasekaran Kaliaperumal

**Affiliations:** 1 The University of Edinburgh, Edinburgh, Scotland, UK; 2 Oxford University Hospitals NHS Foundation Trust, University of Oxford, Oxford, England, UK; 3 NHS Scotland, Edinburgh, Scotland, UK

**Keywords:** COVID-19 pandemic, Neurosurgery, Publication productivity, h-index

## Abstract

**Background:**

Our aim was to determine the impact of the COVID-19 pandemic on the publication productivity of neurosurgeons in the United Kingdom and Republic of Ireland.

**Methods:**

Using bibliometric data we quantified and analysed the academic output of neurosurgeons in England, Scotland, Northern Ireland, Wales, and the Republic of Ireland, between two time periods i.e., January 2017 to December 2019 and January 2020 to March 2022, as a representative capture of the academic climate before and after the start of the COVID-19 pandemic. The consultant neurosurgeons were grouped according to their departments, title, sex, subspecialities and additional research qualifications. Using data charts on Scopus author directory, the total number of publications, citations and h-indices of each neurosurgeon were obtained over the two time periods. The median and mean of these 3 parameters were computed and the median values were analysed and tested for significance using a Mann Whitney-U test according to the groups.

**Results:**

Our analysis conveyed a statistically significant increase (2440 publications and between January 2020 and March 2022 there were 2548 publications p<0.05) in the total number of publications after the start of the COVID-19 pandemic compared to before. There was a statistically significant decrease in the mean number of citations (mean 55.24 vs 57.01, p<0.05), after the start of the COVID-19 pandemic. This trend was observed in both sexes, in authors without an additional MD/PhD and in authors who sub-specialized in neuro-oncology. Overall, there was a significant decrease in H-index after the start of the pandemic compared to before (median h-index:1.00 and 2.00; mean h-index:1.8 and 3.4 respectively).

**Conclusions:**

There appears to be an apparent increase in total number of publications after the start of the COVID-19 pandemic, most authors have registered a reduction in citations and h-indices, suggesting a lower impact and unequal distribution of the abovementioned increase.

## Introduction

Coronavirus disease 2019 (COVID-19), caused by the novel severe acute respiratory syndrome coronavirus 2 (SARS-CoV-2), introduced itself as an infectious acute respiratory infection, however very quickly demonstrated catastrophic extrapulmonary manifestations ^1, 2^. As of October 2022, at least 615 million COVID-19 infections were recorded globally, resulting in 6.5 million fatalities ^3^. Undoubtedly the COVID-19 pandemic has introduced changes in the way we learn, work, interact and plan ^4^.

In light of this, as members of the healthcare staff became more intellectually and emotionally engaged in fulfilling their roles as frontliners in this battle, many of them found it challenging to pursue personal and academic interests outside their clinical environment ^5^. Despite the influx of COVID-19 related articles being published from each specialty, academic institutions and researchers alike would be interested in deciphering the actual impact of COVID-19 on academic productivity, in order to be able to plan and design research better in the future ^6^.

Scientific research and publications significantly contribute to the intellectual, societal and economic growth of a country, as questions are answered or at the least understood better. In 2018, the United Kingdom was ranked the 3 ^rd^ worldwide in terms of number of publications after having published at least 200,000 publications and, in many studies, the UK had cemented its position as a consistent player in contributing to the global scientific publications ^7– 9^. The UK has also always boasted a decent citations-per-publication ratio (CPP), amounting to a relative global publication impact of at least 77% and has always maintained a stable Impact Relative Rank Score (IRRS) at approximately 80% in Medicine ^8, 10^. In 2012, the UK was found to have the highest number of publications per research and development expenditure ^10^. These studies highlight the diligence and prioritization of academic work in the UK.

Hence, we sought to determine whether the COVID-19 pandemic had an impact on the academic productivity of neurosurgeons in the United Kingdom and Republic of Ireland. In this study we have compared the academic output of neurosurgeons in terms of number of publications, based in England, Scotland, Northern Ireland, and Wales as well as the Republic of Ireland. The comparison was made between two timelines before (between January 2017 and December 2019) and after (January 2020 to March 2022) the COVID-19 pandemic. Bibliometric data included the total number of publications and citations, as well as the h-index in the abovementioned stipulated timeframes. With these tools we compared author productivity before and after the start of the COVID-19 pandemic based on author sex, rank, higher research degrees, sub-specialty, and departments.

## Methods

### Study design

***Consultant list and demography*.** A list of neurosurgical departments throughout the UK and Republic of Ireland was obtained from the Society of British Neurological Surgeons (SBNS). The list of consultant neurosurgeons from each department were obtained from their respective NHS hospital staff directories and from individual departmental websites. The details were then confirmed on the UK GMC List of Registered Medical Practitioners and Irish Medical Council. Data was collected for a total of 412 neurosurgeons from 37 neurosurgical departments. Using information from the individual hospital departmental websites or from a broader web search the neurosurgeons were grouped according to their departments, title, sex, subspecialities and further research qualifications (MD or PhD in addition to MBBS and FRCS qualifications).

At the time of writing, the sex of the authors was set according to the self-announced pronouns they had used when addressing themselves in their hospital staff directories, journal articles, and blogs. We recognized that this is a potential limitation, as pronouns do not necessarily tie to a particular sex hence in the future with the help of improved directories, we will devise better methods to be more accurate on their pronouns as we appreciated that the social construct exists on a spectrum. We have refrained from using the term gender as we cannot be certain of authors’ gender using their pronouns. We have adhered to the SAGER guidelines in making our decisions and clarifications ^11^. Similarly, their title and further research qualifications were determined by their affiliation with educational institutions as well as their staff directories. In the United Kingdom and Republic of Ireland, following the basic medical degree i.e., MBBS, in addition to the specialist programmes, doctors can pursue a higher research degree depending on their clinical or academic interests. Both MD and PhD require students to submit a thesis or publication portfolio however the MD programme usually lasts about 2–3 years with an emphasis on clinical practice whereas a PhD programme lasts more than 3 years with an emphasis on laboratory studies ^12^.

Subspecialities were grouped as paediatrics (61), functional/epilepsy (19), peripheral nerve (10), vascular (36), neuro-oncology/skull base (100), general (60), or spinal (126). The numbers in the parentheses correspond to the number of authors from each subspecialty. This order was selected to include the subspecialities that were relatively less represented, given that their papers were related to the subspecialty. For example, when a neurosurgeon was sub-specialized in both paediatrics and spinal neurosurgery, they were considered to be a paediatric neurosurgeon. Likewise, the advent of peripheral nerve surgery has been relatively recent, hence has fewer authors than the other subspecialties ^13^. To ensure that this did not introduce any bias, the150 neurosurgeons who had multiple subspecialties were grouped into a specific subspecialty according to their academic interest as highlighted by their publications in Scopus.

***Patient and Public Information*.** Patients and the public were not involved in this bibliometric analysis. We have only obtained bibliometric data regarding neurosurgeons’ academic productivity from Scopus author directory and would like to thank the platform for aiding with data collection.

***Bibliometric analysis*.** Using Scopus author directory, the total number of publications, citations and h-indices of each neurosurgeon were obtained over the two time periods. The data were recorded manually into Excel spreadsheets according to countries, departments and authors. In order to be able to use the search author function, the first and last names of the author should be known, which were obtained by cross-checking the NHS directory with the departmental website and a broader internet search where necessary. In most cases, the Scopus algorithm accurately differentiated between the authors however, sometimes manual correction was required. A line graph demonstrated the total number of publications and h-indices whilst bar graph demonstrated the total number of citations. We obtained the total number of publications and citations as a sum in the two time periods. We then directly compared the total number of publications and citations within the two different time frames to provide for a valid comparison that reflected the actual situation. The h-index for each individual author was determined from within the two timeframes i.e., January 2017 to December 2019 and January 2020 to March 2022. The Hirsch-index, commonly abbreviated as the h-index, is a bibliometric tool that measures author productivity and citation impact. It is taken as the *n* number of papers that has obtained *n* number of citations for each author ^14^. In this context we have determined the *n* number of papers that has obtained at least *n* number of citations for that particular time frame. Including a bibliometric index in the form of h-index, enables us to normalize the impact of these publication in the international scientific community ^15^. Avoiding the use of further bibliometric tools prevents the introduction of other variables that may introduce irrelevant bias ^14^. Data collection was performed in the first half of 2022; hence the duration of both time frames was slightly different. The period of data collection was carefully selected to ensure that the academic climate was relatively uniform and reflective of the academic climate throughout both time frames, as restrictions and regulations eased significantly towards the second half of 2022, resembling a pre-COVID era. It was our aim to study the true impact of a pandemic on academic productivity.

***Statistical analysis*.** The median and mean of the total number of publications, citations and the median value of the h-index were computed. Considering that the data was not normally distributed we opted to analyse the median values of number of publications, citations and h-indices and tested for significance using non-parametric Mann Whitney-U test for comparison. Significance was set at p<0.05.

## Results

Data were analysed for 412 consultant neurosurgeons (31 female) working in 37 neurosurgical departments across the UK and the Republic of Ireland. This was done after accounting for the overlap in neurosurgical consultant staff in departments in England i.e., the Royal Hallamshire Hospital Department of Neurosurgery and Sheffield Children’s Hospital, in Scotland i.e., Aberdeen Royal Infirmary and Ninewells Hospital and in the Republic of Ireland i.e., Beaumont Hospital and the Children’s University Hospital. In addition, in Scotland, the neurosurgical department at the Western General Hospital Department has recently shifted to the Royal Infirmary of Edinburgh and works in collaboration with the Royal Hospital for Sick Children. The full datasets can be found under underlying data ^16^.

### Publication productivity

Between January 2017 and December 2019 there were 2440 publications and between January 2020 and March 2022 there were 2548 publications (see Table 1). Upon statistical analysis this represented a small but statistically significant increase (p<0.05) in the total number of publications after the start of the COVID-19 pandemic compared to before. This statistically significant increase was seen uniformly in all countries with the sole neurological department in Northern Ireland producing the biggest increase for the country (267%) in total and mean number of publications per year, followed by Republic of Ireland (50%), Wales (38%), Scotland (30%) and England (0.6%) in that order. England recorded the highest mean number of publications per year before and after the start of the pandemic (7.23 and 7.30 respectively) with a total of 2212 publications after the start of the pandemic and 2197 publications before (see Table 2).

**Table 1.** Academic Output, measured as total number of publications, citations and median h-indices stratified by author sex, rank, and higher research degree.

		January 2017–December 2019					January 2020–March 2022				
		Publications		Citations		H-Index	Publications		Citations		H-Index
	No	Total No.	Median	Total No.	Median	Median	Total No.	Median	Total No.	Median	Median
**Sex**											
Male	380	2455	3.00	20685	5.00	2	2567	3.00	20508	6.00	1
Female	31	122	3.00	1433	11.50	2	137	3.50	927	9.50	1
**Rank**											
Mr/Ms	390	1926	3.00	13529	7.00	2	2116	4.00	14391	8.00	1
Professor	21	507	16.00	7062	128.00	9	418	9.00	6067	64.00	3
**MD/PhD**											
Holders	96	1087	6.00	11163	21.50	4	1080	6.00	11051	17.00	2
Non- Holders	315	1334	3.00	9147	6.00	2	1446	3.00	9027	7.00	1

**Table 2.** Academic output measured as total number of publications stratified by country.

Country	January 2017-December 2019		January 2020-March 2022	
	Total Number of Publications	Median Number of Publications	Total Number of Publications	Median Number of Publications
**England**	2197	3.00	2212	3.00
**Scotland**	165	2.00	216	3.00
**Republic** **of Ireland**	43	2.00	58	6.00
**Northern** **Ireland**	6	1.00	22	6.00
**Wales**	29	3.50	40	3.00

There was a statistically significant decrease in the overall total and mean number of citations after the start of the pandemic compared to before (mean 55.24 vs 57.01, p<0.05). However, this trend was not observed individually in all countries, as Scotland and Wales recorded a minor but significant increase in the mean number of citations.

Upon overall analysis there was a statistically significant reduction in the mean and median h-index after the start of the pandemic compared to before (median h-index:1.00 vs 2.00; mean h-index:1.8 vs 3.4). This statistically significant reduction in median h-index was noted especially in Wales, England and Scotland.

### Author sex

**Figure 1.**
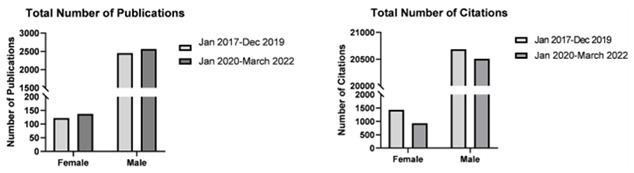
Bar graphs depicting total number of publications by female and male neurosurgeons before (January 2017–December 2019) and after (January 2020–March 2022) the start of the pandemic.

Generally, both female and male neurosurgeons had a statistically significant increase in the total number of publications after the start of the pandemic compared to before (mean number of publications by female neurosurgeons 5.27 vs 4.69 respectively: mean number of publications by male neurosurgeons 7.67 vs 7.22). This was also reflected as a higher median number of publications after the start of the pandemic compared to before. However, female neurosurgeons have recorded fewer citations like their male counterparts after the start of the pandemic as reflected by lower mean and median number of citations ( Figure 1). Ultimately both male and female neurosurgeons registered a statistically significant decrease in h-indices after the start of the pandemic compared to before.

### Author rank and higher research degrees

**Figure 2.**
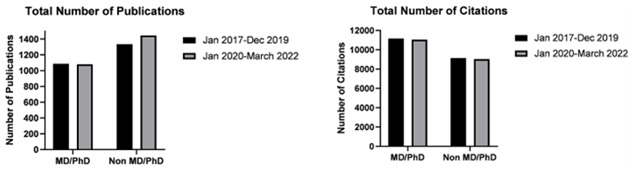
Bar graphs depicting total number of publications by neurosurgeons with and without additional research degrees (MD/PhD), before (January 2017–December 2019) and after (January 2020–March 2022) the start of the pandemic.

In terms of authors with an additional MD or PhD, a small but statistically significant decrease in the number of publications was noted, after the start of the pandemic compared to before, despite recording the same median number of publications (mean number of publications 11.25 vs 11.32, respectively; p<0.05) ( Figure 2). Nevertheless, the trend of reduction in number of citations and a reduction in h-indices after the start of the pandemic was noted in authors with or without higher research degrees alike (see Table 1). 

**Figure 3.**
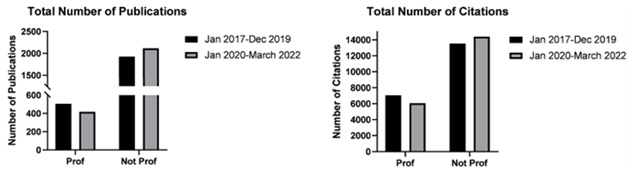
Bar graphs depicting total number of publications by neurosurgeons ranked as Professors and junior authors not ranked as professors, before (January 2017–December 2019) and after (January 2020–March 2022) the start of the pandemic.

Authors ranked as professors registered a statistically significant decrease in total and median number of publications after the start of the pandemic compared to before (total 139 vs 169; median 9.00 vs 16.00 respectively; p<0.05). This also resonated as a decrease in the median number of citations as well as a decrease in h-index. On contrary, the 317 authors with a rank below professors, collectively recorded an increase in total number of publications (median 4.00 vs 3.00 respectively; p<0.05) ( Figure 3). Generally, this group of authors also recorded a significant increase in median number of citations however this did not prevent the reduction in the median h-index, as only a small number of these authors recorded the above-mentioned increases (see Table 1). 

### Author sub-specialty

Neurosurgeons who sub-specialized in neuro-oncology produced a significant increase in the total and median number of publications after the start of the pandemic compared to before. Neurosurgeons who specialized in functional/epilepsy surgery recorded a statistically significant increase in the median number of publications after the start of the COVID-19 pandemic, despite a small but significant decrease in the total number of publications. All other subspecialities recorded a significant decrease in median number of publications and this was also reflected as a reduction in the mean and total number of publications (see Table 3). Peripheral and vascular neurosurgeons generally recorded the greatest decrease in publication count after the start of the pandemic (mean number of publications 6.3 vs 7.5 and 6.4 vs 7.6 respectively). In addition, all sub-specialities recorded a significant decrease in h-indices between January 2020 and March 2022, as generally evident in the reduction in median h-indices in all specialties.

**Table 3.** Academic Output, measured as total number of publications, citations and median h-indices stratified by author sub-specialty before (January 2017–December 2019) and after (January 2020–March 2022) the start of the pandemic.

	January 2017–Decemebr 2019					January 2020–March 2022				
	Publication		Citations		H-Index	Publication		Citations		H-Index
Sub- Specialty	Total Number	Median	Total Number	Median	Median	Total Number	Median	Total Number	Median	Median
Paediatric	407	5.00	3030	13.00	3	398	4.00	3044	10.00	1
Functional	183	3.00	1259	7.00	3	179	6.00	499	6.50	1
Peripheral	75	6.00	200	7.00	3	63	4.50	134	7.00	2
Vascular	237	6.00	1334	8.00	3	199	5.00	1081	10.00	2
Neuro-Oncology	645	5.00	2865	8.50	3	844	5.50	4296	13.00	2
General	417	3.00	6085	9.00	2	394	2.00	5452	4.00	1
Spine	293	2.00	5107	5.00	1	252	2.00	4347	4.00	1

### Department

In England, 11 departments recorded statistically significant increases in the total number of publications after the start of the pandemic compared to before, whereas 8 departments recorded no significant change, and 6 departments recorded a significant decrease. Only 7 departments recorded a statistically significant increase in total number of citations, 12 departments recorded a significant decrease, and 6 departments recorded no significant change. In Scotland, 2 departments reported no significant change in total number of publications, whereas 1 department recorded a significant increase, and 1 department recorded a significant decrease after the start of the pandemic. 2 departments recorded a significant increase in total number of citations and 2 departments recorded no significant change. Similarly in Northern Ireland, the sole department registered no significant change in total number of publications after the start of the pandemic. In the Republic of Ireland one department recorded no significant change, and one department recorded a significant increase in total number of publications as well as citations. In Wales, the sole neurosurgical department recorded no significant change in total number of publications nor in total number of citations.

The top 5 neurosurgical departments before the start of the pandemic were from the The National Hospital for Neurology & Neurosurgery Victor Horsley, Addenbrooke’s Hospital, King’s College Hospital, Oxford Radcliffe NHS Trust and Hope Hospital Department of Neurosurgery. After the start of the pandemic the top 5 neurosurgical departments were from the same hospitals however in a different order: King’s College Hospital, The National Hospital for Neurology & Neurosurgery Victor Horsley, Addenbrooke’s Hospital, Oxford Radcliffe NHS Trust and Hope Hospital Department of Neurosurgery. In the top 3, the former recorded a significant decrease in total number of citations whereas the latter two departments recorded a significant increase in total number of citations. All 5 departments are based in England (see Table 4).

**Table 4.** Academic Output, measured as total number of publications, median number of publications and h-indices stratified by departments before (January 2017–December 2019) and after (January 2020–March 2022) the start of the pandemic.

			Jan 2017–December 2019			January 2020–March 2022		
			Publications		H-index	Publications		H-index
Country	Departments	No.	Total	Median	Median	Total	Median	Median
England	Addenbrooke’s Hospital, Neurosurgery Unit	18	298	5.00	4.50	305	6.00	2.00
	Royal Preston Hospital, Department of Neurosurgery	12	21	0.50	0.00	35	1.00	0.50
	Hope Hospital Department of Neurosurgery	27	125	5.00	2.00	127	5.00	1.00
	Royal Manchester Children's Hospital Oxford Road	7	25	2.50	2.50	20	2.00	1.00
	Oxford Radcliffe NHS Trust Department of Neurosurgery	17	244	6.00	4.00	214	7.00	2.00
	North Bristol NHS Trust Southmead Hospital	16	71	3.00	2.00	43	1.50	0.00
	Bristol Royal Hospital for Children	3	10	2.00	2.00	8	3.00	1.00
	University Hospitals Plymouth NHS Trust, Department of Neurosurgery	10	43	5.00	1.00	31	3.50	0.00
	Royal Hallamshire Hospital Department of Neurosurgery, Sheffield Children’s Hospital	20	81	5.00	2.00	85	4.00	1.00
	Queen’s Medical Centre Department of Neurosurgery	18	56	3.00	2.00	65	4.50	2.00
	Queen Elizabeth Neuroscience Centre Department of Neurosurgery	20	115	2.00	2.00	127	3.00	1.50
	Birmingham Children’s Hospital Department of Neurosurgery	2	8	4.00	3.00	6	3.00	2.00
	North Staffordshire Hospital Trust Department of Neurosurgery	16	17	2.00	2.00	27	3.00	1.50
	Walsgrave Hospital Department of Neurosurgery	14	41	1.50	1.00	42	1.50	0.00
	Wessex Neurological Centre, Department of Neurosurgery, Southampton General Hospital	14	79	3.00	3.00	81	4.00	1.00
	Hull Royal Infirmary Department of Neurosurgery	14	23	1.50	1.00	23	2.00	1.00
	The General Infirmary at Leeds Department of Neurosurgery	17	111	5.50	6.50	111	6.50	1.00
	Charing Cross Hospital Department of Neurosurgery	15	49	5.00	2.00	40	2.00	1.00
	St Bartholomew’s and Royal London Hospital Department of Neurosurgery	7	15	3.00	2.00	14	1.00	0.00
	Essex Neurological Centre Department of Neurosurgery	13	33	3.00	1.00	26	2.00	0.50
	King’s College Hospital Department of Neurosurgery	24	250	7.00	3.00	325	8.00	2.50
	Hurstwood Park Neurological Centre Department of Neurosurgery The Princess Royal Hospital	3	12	1.00	1.00	9	2.00	0.00
	Atkinson Morely Wing, Department of Neurosurgery, St George's Hospital	13	36	1.00	0.00	31	2.00	1.00
	The National Hospital for Neurology & Neurosurgery Victor Horsley, Department of Neurosurgery	24	347	10.50	6.00	320	10.50	2.50
	Great Ormond Street Hospital for Children Department of Neurosurgery	6	102	16.00	9.50	116	21.50	3.00
Scotland	Western General Hospital Department of Clinical Neurosciences, Royal Hospital for Sick Children	13	106	6.00	19.00	157	9.00	6.00
	Institute of Neurological Sciences, University Department of Neurosurgery, Southern General Hospital NHS Trust	5	9	2.00	1.00	13	3.00	1.00
	Greater Glasgow and Clyde	15	27	1.50	1.00	26	2.00	0.50
	Aberdeen Royal Infirmary Department of Neurosurgery, Ninewells Hospital And Medical School Dept of Neurosurgery	5	23	5.00	3.00	20	6.00	1.50
Northern Ireland	Royal Victoria Hospital Department of Neurosurgery	4	6	1.50	1.00	22	5.00	1.00
Republic of Ireland	Beaumont Hospital Department of Neurosurgery, The Children's University Hospital	10	36	3.50	1.00	44	5.50	1.50
	Cork University Hospital Department of Neurosurgery	4	7	2.00	1.00	14	4.00	1.00
Wales	University Hospital of Wales Department of Neurosurgery	6	29	3.50	2.50	40	3.00	0.50

In terms of h-indices, generally most departments across the 4 countries recorded significant reductions in median h-indices after the start of the pandemic compared to before.

## Discussion

It is evident that over the same span of 3 years authors had more publications after the pandemic started than prior to the commencement of the COVID-19 pandemic and this was subsequently followed by a decrease in the number of citations. However, the h-index reduced, and this highlights the strength of the bibliometric tool, as it depicts that the above-mentioned increases were not seen uniformly by all authors and in fact many authors were unable to sustain their academic productivity after the start of the pandemic.

Neurosurgeons with an MD or PhD and professors have in the past registered consistent publication productivity. Nevertheless, after the start of the pandemic, they have recorded significant decreases in total number of publications. Whilst the number of publications is still higher than junior authors without MD or PhD, this dip truly reflects the negative impact of the pandemic on seasoned publications by authors.

There can be a number of reasons for the decrease in h-index in neurosurgery ^5^. Firstly, there was a clear redirection of funding and resources after the start of the pandemic. Considering the sour impact of lockdown on the economy, research funding by governmental bodies, public and private institutions have become more cautious ^5, 17^. Undoubtedly, the priority had been to cater to the influx of research articles related to COVID-19 ^18^. Studies have shown that the UK had allocated at least £25 million for 26 different projects to understand the nature and behaviour of COVID-19 aiming to find a solution to beat the pandemic. It was also noted that the approach taken by the Medical Research Council (MRC) and National Institute of Health Research (NIHR) included much more flexibility and agility in allocating funds as long as the projects could offer any valuable contribution. This resonated the need to act swiftly and rapidly amidst the evolving pandemic ^19^.

Apart from this, research staff shortages were prominent due to clinical priorities, family priorities, travel restrictions, COVID-19 infection or quarantine ^5^. This served as a major impediment to data collection, collaboration meetings hence impacted data analysis and discussion ^20^. Collectively, these have led to stalling of projects as they were unable to proceed according to plan ^21^.

Many researchers have admitted that it has been difficult to recruit research participants, particularly non-COVID patients ^20^. Certain specialities were affected more than others as resources and staff members were directed toward the frontline or administration of the battle against the pandemic. This is evident as most sub-specialities in our study recorded a decrease in total number of publications. As seen in our study, authors practising as peripheral and vascular neurosurgeons both recorded a decrease in publication count by 16% after the start of the pandemic. During the pandemic, consultant neurosurgeons and their teams had to devise newer management plans after postponing elective procedures, and now as the effects of the pandemic are weaning down, surgeons have to clear the backlog of cases ^22, 23^. Hence the extent of the impact on publication productivity may not have been completed yet.

The impact of the pandemic on publication productivity is an important lesson and reminder on how this can affect the scientific infrastructure and neurosurgery in this context. Healthcare research has a cardinal role in understanding, reflecting, and discovering the fundamentals of clinical practice as progression is only made when exploring the unknown. The inquisitive intent for identifying shortcomings and introducing changes must be protected and the reason to do so was highlighted by this pandemic, as we experienced different phases of setbacks and success depending on our cumulative knowledge at different points of our battle with COVID-19 ^24^. As mentioned by Muller *et al*., academic productivity of clinicians is not only dependent on the authors’ proficiency but also the scientific infrastructure that is in turn determined by the prevailing epidemiological, economical and healthcare factors ^25^. Countries with a high Healthcare Access and Quality index (HAQ-index), high number of physicians per 1000 people, high healthcare spending per capita and high GDP positively correlated with publication productivity even after the start of the pandemic. In fact, with a stronger foundation, certain factors such as a good division of labour in research and greater COVID-19 infection rates (presenting more opportunity for data collection in COVID-19 related studies) were used as leverage points during the pandemic to spur publication count growth to a rate higher than before the start of the pandemic. As reflected in our data analysis, the United Kingdom did depict an increase in total number of publications after the start of the pandemic due to the above-mentioned reasons. However, invariably there is still room for improvement, and as noted from our study, being able to maintain the h-index should be the aim.

On the flip side, we do note that the previously mentioned disparity in academic neurosurgery is improving as both male and female neurosurgeons recorded an increase in number of publications, despite the increased personal responsibilities introduced by lockdown ^26^. In our study it was seen that the publication count varied in the different subspecialties, with female neurosurgeons in Neuro-oncology registering at least 100 publications before and after the start of the pandemic, over the span of 3 years. This number and increase was not seen in the other subspecialties calling for more efforts for better representation of female neurosurgeons in the different subspecialties ^27^. Our results have just depicted their publication productivity; however, it is important to extrapolate this to the complex academic nexus as more female neurosurgeons are being established as senior authors and are being provided mentorships, sponsorships, and a position in the editorial boards. In fact, in these studies, it was noted that female authors with female supervisors were more likely to publish their articles and this is a point to explore further and reflect on ^27, 28^.

Another point of note is the contribution by junior authors to academic neurosurgery. As discerned in our data and results, senior authors still led the ranks in terms of total number of individual publications however, the majority of increase in total number of publications can be attributed to junior authors. This implies good mentorship by senior authors and the strength of efforts by medical schools and academic programmes to prime junior authors into developing their academic proficiency ^29^. With good access to data collection, junior authors are continuously devising more innovative approaches to data collection as highlighted by the increase in total number of publications despite lockdown measures. This, coupled with increased review rate by journals, would be fruitful to the academicians ^30^.

## Conclusion

There appears to be an apparent increase in total number of publications after the start of the COVID-19 pandemic, most authors have registered a reduction in citations and h-indices, suggesting a lower impact and unequal distribution of the abovementioned increase. Senior authors recorded a reduction in number of publications, citations, and h-indices. This truly highlights the negative impact that the COVID-19 pandemic has had on quantity and quality of academic productivity.

Protecting our scientific infrastructure and ensuring that we have learnt our lessons in preparing for any such future challenges should be considered. Academic productivity can be a good indicator of how well the academic clinicians are coping with the prevailing healthcare and economic challenges, as quite infrequently the issue stems from being unable to juggle clinical, academic, personal, and social responsibilities. Solutions can range from establishing a good proportion of non-clinicians and junior doctors helping to increase the number of physicians and support for academic clinicians where costs permit. A positive outlook is seen from our study, as junior authors are making their mark.

It is also evident that female neurosurgeons were able to publish more despite the challenges imposed by the pandemic. This trend should hopefully continue post COVID-19 pandemic to aid equality in neurosurgery.

## Limitations

There are limitations to our study, firstly considering the retrospective nature of our data collection, the accuracy of data extraction from the available databases can be limited by suboptimal data input. The two timelines compared are not of equal length. Data collection was performed in March 2022. This was partly due to the limitation of our source of data, in which we were unable to select the publications and citations for selected months of the year to ensure that both timeframes were of equal length. In addition, papers written are usually published a year or two after writing. Nevertheless, we aimed to study the impact of COVID-19 on publication productivity, hence proceeded with the two timeframes that will be true representatives of the pre-COVID an post-COVID era, bearing in mind that the academic environment in the later stages of 2022 was rapidly returning to the pre-COVID era. Furthermore, the h-indices were specifically selected for each period, ensuring that they were only derived from publications and citations in those two timeframes.

Another limitation that we would like to highlight is due to the large volume of data analysed under multiple different categories, there is a potential for inflated false positives. In the future, we would apply the Bonferroni correction to avoid this. In this paper, we were able to accurately categorize the data as can be viewed in our data tables and we ensured that under each category of dataset we did not run multiple or layered statistical test to reduce the chance of false positive inflation. We also had a large number of multiple comparisons hence application of the Bonferroni correction may lead to a very high rate of false negatives. 

In addition, for this study we did not explore the type of research projects published by the authors and it will be valuable to explore this in future studies. Similarly, we did not distinguish between COVID-19 and non-COVID-19 articles by the authors, and it is understood that certain factors such as an increase in COVID-19 patients means a more chance at data collection may promote a boost in the publication count of COVID-19 related articles that maybe reflected on total publication count. The impact factor of the journals published may also have changed over time and this could impact for difference in h-indices.

As previously mentioned at the time of writing, the sex of the authors was set according to the pronouns they had used when addressing themselves in their hospital staff directories, journal articles, and where available blogs. We recognized that this is a potential limitation, as pronouns do not necessarily tie to a particular sex and in the future with the help of improved and accurate directories, we will devise better methods to be more accurate on their identifying an author’s socially constructed role as we appreciate that the construct exists on a spectrum.

## Data Availability

Figshare: Neurosurgical Publications Data Tables. https://doi.org/10.6084/m9.figshare.24418171.v2 ^16^ This project contains the following underlying data: England Neurosurgical Publications Data Table.csv Scotland Neurosurgical Publications Data Table.csv Wales Neurosurgical Publications Data Table.csv Republic of Ireland Neurosurgical Publications Data Table.csv Neurosurgical Departments and Publications .csv Data are available under the terms of the Creative Commons Attribution 4.0 International license (CC-BY 4.0).
