# Supplementary material for: Has COVID-19 affected the publication productivity of neurosurgeons in UK and Republic of Ireland? A bibliometric analysis
Source: Health Open Res. 2024 Aug 12;5:39. Originally published 2023 Dec 15. [Version 2] doi: 10.12688/healthopenres.13445.2 (PMC11736354; doi:10.12688/healthopenres.13445.2)
Supplement: Peer review history

# Reviewer report: Reviewer response for version 2

**DOI:** 10.21956/healthopenres.14749.r29190

**Published:** 2024-12-17

**Authors:** Gaurav Mittal, Prashanth A

**Affiliations:** 1 Mahatma Ghandhi Institute of Medical Sciences, Wardha, India; 2 Mahatma Gandhi Institute Sciences, Maharashtra, India

Firstly, I appreciate the unique method of the study of scientometrically analysing the data through the author directory and not the articles. 

**The major flaws that I see are:**

 1. If an author based search was done, why was only SCOPUS searched upon, there are many journals indexed with other databases like PubMed and Web of Science being the important ones in medical sciences. This methodology might have missed potential articles. 

 I would strongly suggest including these databases by searching the author's name (as author directories are unavailable, unlike SCOPUS).

 2. I am afraid, this review misses out on an important aspect of increasing research collaborations after COVID 19 [1]. 

 3. I would also like to understand how this study takes the research field forward and what the research question exactly was and the need for this study. 

 4. Considering COVID 19 as a reason for surged publications can also be confounding.

 5. Another important aspect missing is the type of publications (Research articles/Review articles), since an author based search is done it would be beneficial to understand the same. 

 6. The methodology is again flawed as pre COVID 2 Years are taken into consideration and post COVID 2 years and 3 months which again might lead to a huge confounding variable. 

 7. While there is an increase in total publications, a decrease in citations and h-index is observed. The study does not provide a detailed explanation for this discrepancy. Discuss this to move the field forward and for future studies.

Is the work clearly and accurately presented and does it cite the current literature?

Yes

If applicable, is the statistical analysis and its interpretation appropriate?

I cannot comment. A qualified statistician is required.

Are all the source data underlying the results available to ensure full reproducibility?

Yes

Is the study design appropriate and is the work technically sound?

Partly

Are the conclusions drawn adequately supported by the results?

No

Are sufficient details of methods and analysis provided to allow replication by others?

Partly

Reviewer Expertise:

Neurosurgery

We confirm that we have read this submission and believe that we have an appropriate level of expertise to confirm that it is of an acceptable scientific standard, however we have significant reservations, as outlined above.

## References

[1] Kaneko T, Saeki S. Research Collaborations in the Post-COVID Era. JMA J. 2023 Jul 14;6(3):358-359. doi: 10.31662/jmaj.2023-0034. Epub 2023 May 12. PMID: 37560361; PMCID: PMC10407446. 10.31662/jmaj.2023-0034PMC1040744637560361

# Reviewer report: Reviewer response for version 2

**DOI:** 10.21956/healthopenres.14749.r28751

**Published:** 2024-10-10

**Authors:** Gokhan Tazegul

**Affiliations:** 1 Marmara University, Istanbul, Turkey

The paper provides a clear bibliometric overview regarding the impact of the COVID-19 pandemic on the publication productivity of neurosurgeons in the United Kingdom and the Republic of Ireland. The methods used, including analyzing bibliometric data and grouping consultant neurosurgeons based on various criteria, are well-defined and comprehensive. The results are poorly presented; revision notes can be found below. The discussion and conclusions are drawn from and are supported by the results; the unequal distribution of the increase in publications is thought-provoking and warrants further discussion. I recommend a revision of the results for further review.

 Revision notes: 

 Results: Authors should provide exact p values rather than p<0.05. Authors should also give either range (min-max) or interquartile range (25th-75th) when reporting medians.

 "There was a statistically significant decrease in the overall total and mean number of citations after the start of the pandemic compared to before (mean 55.24 vs 57.01, p<0.05)." When comparing medians, do not provide means; report medians. 

 "Generally, both female and male neurosurgeons had a statistically significant increase in the total number of publications after the start of the pandemic compared to before (mean number of publications by female neurosurgeons 5.27 vs 4.69 respectively: mean number of publications by male neurosurgeons 7.67 vs 7.22). " Similar errors, report exact p values, show differences in Figure 1, report medians when using Mann-Whitney U test.

 "In terms of authors with an additional MD or PhD, a small but statistically significant decrease in the number of publications was noted." Report exact values and report exact p values. Do not discuss; only report values in Results (no need to say "small but statistically significant").

 Revise whole Results accordingly.

Is the work clearly and accurately presented and does it cite the current literature?

Yes

If applicable, is the statistical analysis and its interpretation appropriate?

Partly

Are all the source data underlying the results available to ensure full reproducibility?

Yes

Is the study design appropriate and is the work technically sound?

Yes

Are the conclusions drawn adequately supported by the results?

Yes

Are sufficient details of methods and analysis provided to allow replication by others?

Yes

Reviewer Expertise:

Bibliometrics and scientometrics

I confirm that I have read this submission and believe that I have an appropriate level of expertise to confirm that it is of an acceptable scientific standard, however I have significant reservations, as outlined above.

# Reviewer report: Reviewer response for version 2

**DOI:** 10.21956/healthopenres.14749.r28717

**Published:** 2024-09-09

**Authors:** Antica Culina

**Affiliations:** 1 Ruder Boskovic Institute, Zagreb, Croatia

Some of my previous comments were not addressed:

1. Are the observed effects just time trends (i.e. would have happened even without covid) or are reflecting some COVID relate impacts. My suggestion was to add another, earlier pre covid period to the analysis. In that case if we just see that there is e.g. an increase in number pf publications over all three periods, then this is likely not due to covid itself, but just a general time-trend of increase in the number of publications per/year. If not changed in the analysis, it should be at least clearly highlighted in: a) discussion, where it should be clearly stated that this could be just a general time trend and not a covid influence. B) limitations.
2. Add to the Discussion or limitations that Given that publications do not happen immediately after data collection (in my experience it takes at least one year, and generally two years before something gets published). If this is so, then we would expect the impact of COVID-19 to be reflected in the number of publications not in the period 2020-2022 but in 2023-2034.
3. Add to the Methods that data analysis was conducted in Prism.

Is the work clearly and accurately presented and does it cite the current literature?

Partly

If applicable, is the statistical analysis and its interpretation appropriate?

Partly

Are all the source data underlying the results available to ensure full reproducibility?

Yes

Is the study design appropriate and is the work technically sound?

Partly

Are the conclusions drawn adequately supported by the results?

Partly

Are sufficient details of methods and analysis provided to allow replication by others?

Partly

Reviewer Expertise:

ecology, meta-research, open data, meta-analysis

I confirm that I have read this submission and believe that I have an appropriate level of expertise to state that I do not consider it to be of an acceptable scientific standard, for reasons outlined above.

# Reviewer report: Reviewer response for version 1

**DOI:** 10.21956/healthopenres.14532.r27718

**Published:** 2024-01-31

**Authors:** Antica Culina

**Affiliations:** 1 Ruder Boskovic Institute, Zagreb, Croatia

In this article, authors aim to evaluate the impact of the COVID-19 pandemic on the number of publications, and a couple of measures that should capture the wider impact of research (citation, h-index). While I find the topic of the article interesting, and valuable to the intended audience, I have several concerns when it comes to data collection, analysis and thus the interpretation of the results.

 First, data were collected in two time periods, which means it is impossible to determine whether detected changes in variables of interest were just the general time-trends, rather than reflecting the impact of the pandemic. I would suggest collecting data for at least one (and preferably several) time periods prior to the pandemic. This would allow for stronger inferences about the impact of the pandemics.

 Second, the same dataset was analyzed many times, and the issues with this is the potential for inflated false positives (e.g. see here ref[1].) I would suggest considering alternative ways to analyze the data (or correct for multiple tests on the same dataset, as suggested in Ranganathan et al. or elsewhere).

 Apart from the two main concerns I highlight above, I have several other suggestions/comments

1. In the abstract, I would suggest putting the values rather than just stating something was statistically significant. Statistical significance does not tell anything about the size of the effect.
2. Given that publications do not happen immediately after data collection (in my experience it takes at least one year, and generally two years before something gets published). If this is so, then we would expect the impact of COVID-19 to be reflected in the number of publications not in the period 2020-2022 but in 2023-2034. I believe this is something worth discussing
3. I did not understand how were subspecialties treated if a person had two or more of these. If I understood it correctly, in this case the subspeciality with more of other people belonging to was selected. In how many cases has this happened? Could this decision affect the related analysis and results?
4. I am also unclear about how was h-index calculated. Was it calculated only for those publications published within the selected timeframe, and for their citations only during that time-frame?
5. In what software were statistical tests preformed?

Is the work clearly and accurately presented and does it cite the current literature?

Partly

If applicable, is the statistical analysis and its interpretation appropriate?

Partly

Are all the source data underlying the results available to ensure full reproducibility?

Yes

Is the study design appropriate and is the work technically sound?

Partly

Are the conclusions drawn adequately supported by the results?

Partly

Are sufficient details of methods and analysis provided to allow replication by others?

Partly

Reviewer Expertise:

ecology, meta-research, open data, meta-analysis

I confirm that I have read this submission and believe that I have an appropriate level of expertise to state that I do not consider it to be of an acceptable scientific standard, for reasons outlined above.

## References

[1] : Common pitfalls in statistical analysis: The perils of multiple testing. *Perspect Clin Res* .2016;7(2) : 10.4103/2229-3485.179436 106-7 10.4103/2229-3485.179436 27141478 PMC4840791

# Response

**Published:** 2024-02-19

**Authors:** Hariss G Paremes Sivam

**Affiliations:** Department of Clinical Neurosciences, The University of Edinburgh, Edinburgh, Scotland, UK

**In this article, authors aim to evaluate the impact of the COVID-19 pandemic on the number of publications, and a couple of measures that should capture the wider impact of research (citation, h-index). While I find the topic of the article interesting, and valuable to the intended audience, I have several concerns when it comes to data collection, analysis and thus the interpretation of the results.   First, data were collected in two time periods, which means it is impossible to determine whether detected changes in variables of interest were just the general time-trends, rather than reflecting the impact of the pandemic. I would suggest collecting data for at least one (and preferably several) time periods prior to the pandemic. This would allow for stronger inferences about the impact of the pandemics.**

**Response: **We agree to this comment, and will ensure that we will address the potential impact of time-trends on the publication indices, in our upcoming work. We attempted to limit this impact by studying the publication and averaging the values to a set time frame of 3 years in both periods.  

**Second, the same dataset was analyzed many times, and the issues with this is the potential for inflated false positives (e.g. see here ref[1].) I would suggest considering alternative ways to analyze the data (or correct for multiple tests on the same dataset, as suggested in Ranganathan et al. or elsewhere).**

**Response: **We understand that this maybe a limitation of our paper and we will employ the use of the Bonferroni correction as suggested by Ranganathan et al., to ensure that this has been addressed in our future work. In this paper, we were able to accurately categorize the data as can be viewed in our data tables and we ensured that under each category of dataset we did not run multiple or layered statistical test to reduce the chance of false positive inflation. We also had a large number of multiple comparisons hence application of the Bonferroni correction may lead to a very high rate of false negatives.

**In the abstract, I would suggest putting the values rather than just stating something was statistically significant. Statistical significance does not tell anything about the size of the effect.**

**Response:** This has been added.

**Given that publications do not happen immediately after data collection (in my experience it takes at least one year, and generally two years before something gets published). If this is so, then we would expect the impact of COVID-19 to be reflected in the number of publications not in the period 2020-2022 but in 2023-2034. I believe this is something worth discussing**

**Response: **We understand this as a limitation of our study with the time frame. We aimed to capture the prevailing academic environment during the two mentioned time-frames and employed the h-index provide a cumulative picture to eliminate any biasness due to time-trends.

**I did not understand how were subspecialties treated if a person had two or more of these. If I understood it correctly, in this case the subspeciality with more of other people belonging to was selected. In how many cases has this happened? Could this decision affect the related analysis and results?**

**Response: **This has occurred in approximately 150 neurosurgeons that we studied. During the time of data collection, we grouped them under the specialty that their academic interest was this to make the comparison valid. We have now highlighted this in the paper.

**I am also unclear about how was h-index calculated. Was it calculated only for those publications published within the selected timeframe, and for their citations only during that time-frame?**

**Response:** We aimed for the h-index to reflect the number of publications and citations for the two time-frames alone. This was done to normalize the impact of these publication in the international scientific community and provide a cumulative picture to eliminate any biasness due to time-trends.

**In what software were statistical tests preformed?**

**Response:** Prism

# Reviewer report: Reviewer response for version 1

**DOI:** 10.21956/healthopenres.14532.r27727

**Published:** 2024-01-17

**Authors:** Mandara M. Harikar

**Affiliations:** 1 Dept. of Neuroscience, Monash University, Clayton, Victoria, Australia

Thank you for the opportunity to review this manuscript. In this study, the authors present a detailed analysis of neurosurgical research productivity before and after the onset of the pandemic. They found that while the total number of publications rose during the pandemic, the number of citations and H-indices of authors actually fell.

 Overall, the paper is very well written and convincing.

 While it's well known that all research activity proliferated during the pandemic, it would be interesting to know how many of these papers were on COVID-19 or COVID-19 related complications, and not strictly on neurosurgery *per se.*

 It would also be nice to see the goals of this research explained in the Introduction - what are the potential scientific or medical implications of this research? What is the end-goal of your study? 

 How did you know the data was not normally distributed? (A brief explanation/mention of tests will suffice)

 It's impossible to reflect on the reasons for fall in h-indices without knowing **what **the newly published research was about. Without knowing what researchers were publishing about before and after a pandemic, it's hard to comprehend why the h-index still fell - possibly because most neurosurgical researchers did not hop on the COVID-19 research bandwagon, or, because the world has moved on from COVID-19 research, resulting in a few number of articles being cited from that period? The absence of topic-wise analysis is a definite limitation of this study.

Is the work clearly and accurately presented and does it cite the current literature?

Yes

If applicable, is the statistical analysis and its interpretation appropriate?

Yes

Are all the source data underlying the results available to ensure full reproducibility?

Yes

Is the study design appropriate and is the work technically sound?

Yes

Are the conclusions drawn adequately supported by the results?

Yes

Are sufficient details of methods and analysis provided to allow replication by others?

Yes

Reviewer Expertise:

Clinical trials, trial design, neurosurgery, neuroscience, public health

I confirm that I have read this submission and believe that I have an appropriate level of expertise to confirm that it is of an acceptable scientific standard.
